# Supplementary material for: Vibrational spectroscopy analysis of ligand efficacy in human M2 muscarinic acetylcholine receptor (M2R)
Source: Commun Biol. 2021 Nov 23;4:1321. doi: 10.1038/s42003-021-02836-1 (PMC8635417; doi:10.1038/s42003-021-02836-1)
Supplement: Supplementary file 2 — Supplementary Information [file 42003_2021_2836_MOESM2_ESM.pdf]

Supplementary Information

Vibrational spectroscopy analysis of ligand efficacy in human M_2 muscarinic acetylcholine receptor (M_2R)

Kota Katayama[*,†,1,2,3], Kohei Suzuki[†,1], Ryoji Suno[4], Ryoji Kise[5], Hirokazu Tsujimoto[6], So Iwata[6], Asuka Inoue[5], Takuya Kobayashi[4,7], and Hideki Kandori[*1,2]

[1]Department of Life Science and Applied Chemistry, Nagoya Institute of Technology, Showa-ku, Nagoya 466-8555, Japan

[2]OptoBioTechnology Research Center, Nagoya Institute of Technology, Showa-ku, Nagoya 466-8555, Japan

[3]PRESTO, Japan Science and Technology Agency, 4-1-8 Honcho, Kawaguchi, Saitama 332-0012, Japan

[4]Department of Medical Chemistry, Kansai Medical University, Hirakata 573-1010, Japan

[5]Graduate School of Pharmaceutical Sciences, Tohoku University, Sendai, Miyagi 980-8578, Japan

[6]Department of Cell Biology and Medical Chemistry, Graduate School of Medicine, Kyoto University, Kyoto 606-8501, Japan

[7]Japan Agency for Medical Research and Development, Core Research for Evolutional Science and Technology (AMED-CREST), Chiyoda-ku, Tokyo 100-0004, Japan

[†]These authors contributed equally

[*]Correspondence and requests for materials should be addressed to K.K. (email: katayama.kota@nitech.ac.jp), H.K. (email: kandori@nitech.ac.jp)

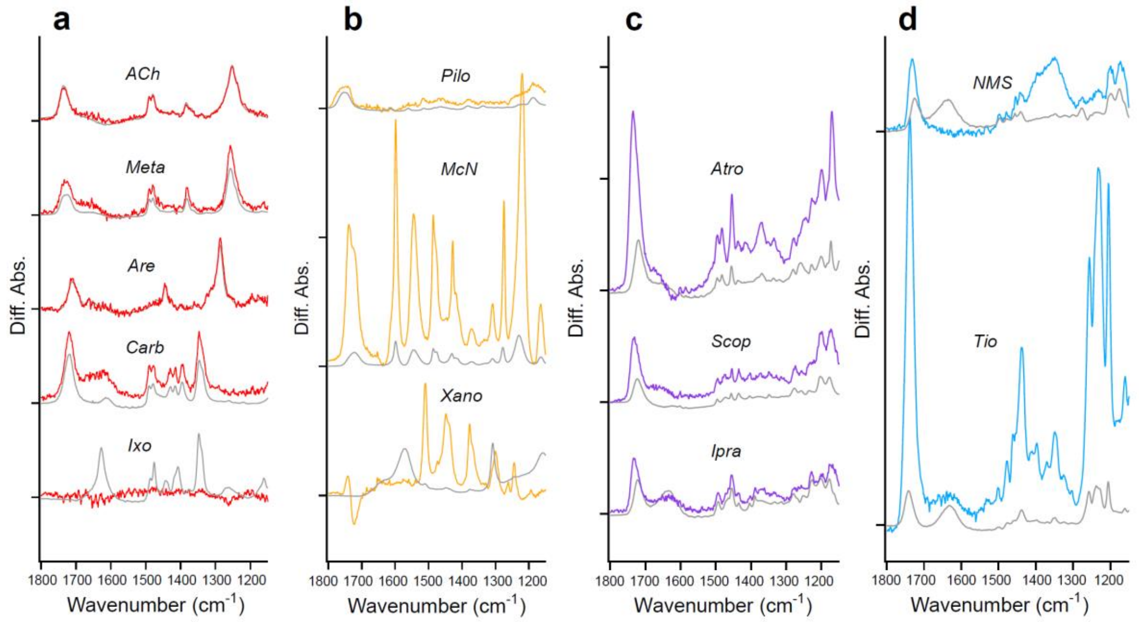

Supplementary Figure 1. Comparison of IR absorption of each ligand. (a) Red and grey lines represent agonist binding-induced difference ATR-FTIR spectra with empty liposome but without protein and without both protein and empty liposome in the 1800-1150 cm^{-1}, respectively. One division of the y-axis corresponds to 0.0003 absorbance unit. (b) Orange and grey lines represent partial agonist binding-induced difference ATR-FTIR spectra with empty liposome but without protein and without both protein and empty liposome in the 1800-1150 cm^{-1}, respectively. One division of the y-axis corresponds to 0.001 absorbance unit. (c) Purple and grey lines represent antagonist binding-induced difference ATR-FTIR spectra with empty liposome but without protein and without both protein and empty liposome in the 1800-1150 cm^{-1}, respectively. One division of the y-axis corresponds to 0.0005 absorbance unit. (d) Cyan and grey lines represent agonist binding-induced difference ATR-FTIR spectra with empty liposome but without protein and without both protein and empty liposome in the 1800-1150 cm^{-1}, respectively. One division of the y-axis corresponds to 0.0015 absorbance unit.

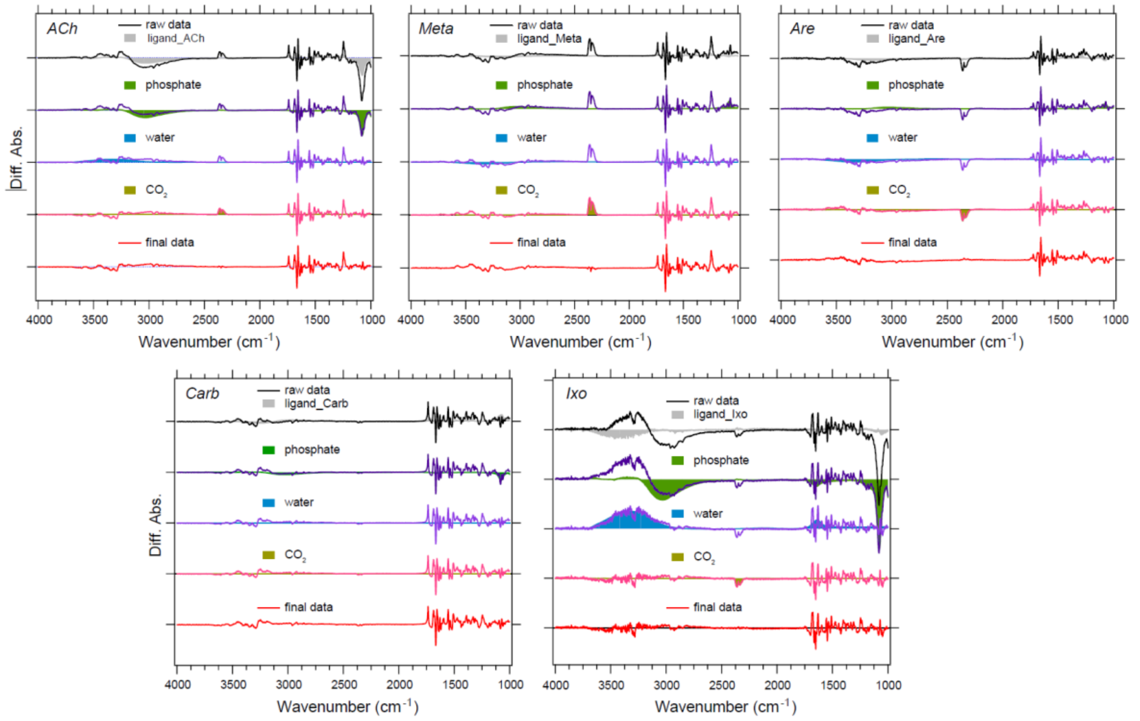

Supplementary Figure 2. The agonist binding-induced difference ATR-FTIR spectra in the 4000-1000 cm^{-1} region. The absorption of unbound ligands (shaded grey), the absorption of phosphate buffer (shaded green), the absorption of water (shaded cyan) and the absorption of CO_2 (shaded yellow) are subtracted for spectral correction.

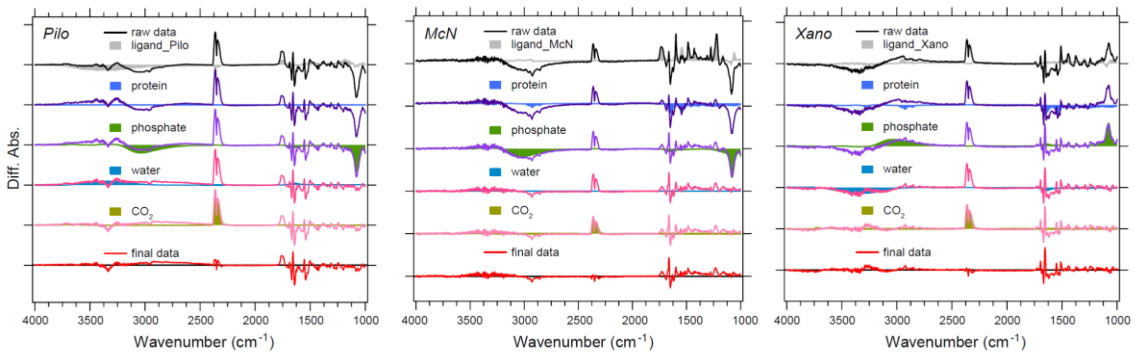

Supplementary Figure 3. The partial agonist binding-induced difference ATR-FTIR spectra in the 4000-1000 cm^{-1} region. The absorption of unbound ligands (shaded grey), the absorption of phosphate buffer (shaded green), the absorption of water (shaded cyan) and the absorption of CO_2 (shaded yellow) are subtracted for spectral correction.

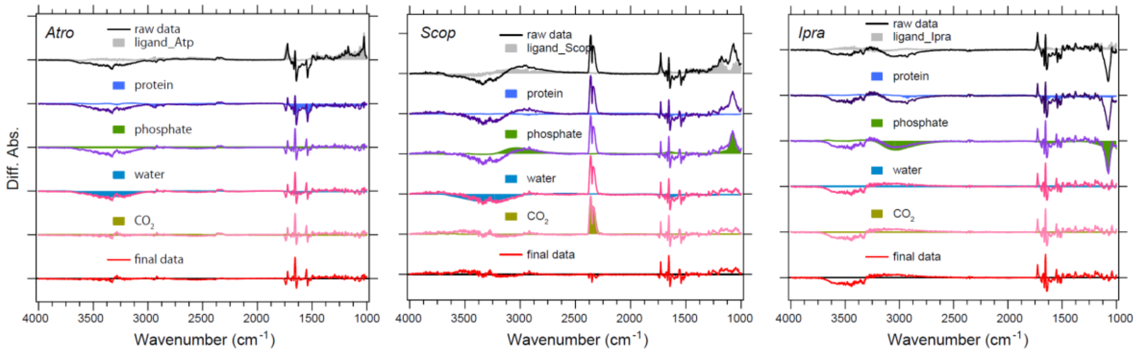

Supplementary Figure 4. The antagonist binding-induced difference ATR-FTIR spectra in the 4000-1000 cm^{-1} region. The absorption of unbound ligands (shaded grey), the absorption of phosphate buffer (shaded green), the absorption of water (shaded cyan) and the absorption of CO_2 (shaded yellow) are subtracted for spectral correction.

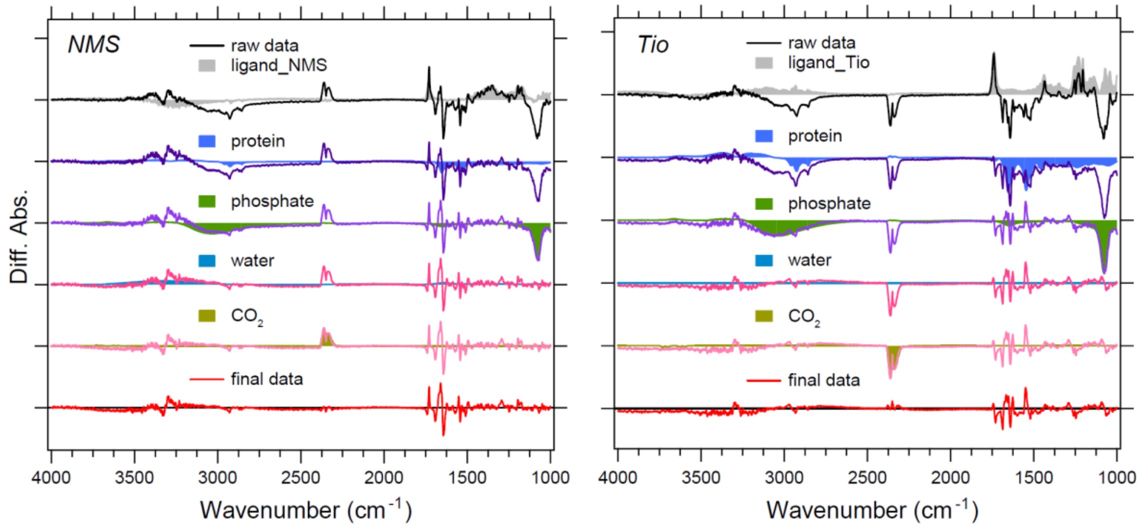

Supplementary Figure 5. The inverse agonist binding-induced difference ATR-FTIR spectra in the 4000-1000 cm^{-1} region. The absorption of unbound ligands (shaded grey), the absorption of phosphate buffer (shaded green), the absorption of water (shaded cyan) and the absorption of CO_2 (shaded yellow) are subtracted for spectral correction.

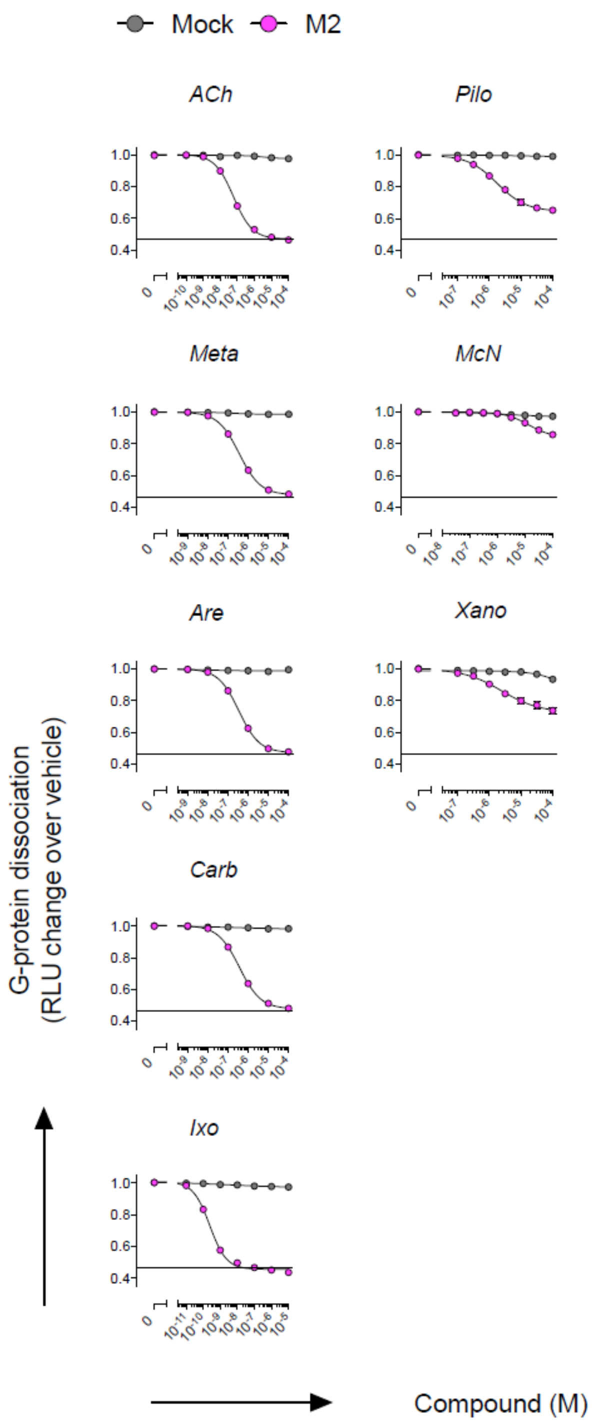

Supplementary Figure 6. Concentration-response curves of G_i activation in NanoBiT G-protein dissociation assay of M_2R. Symbols and error bars represent mean and SEM, respectively, of 5 or 10 (shown in parenthesis) independent experiments with each performed in duplicate.

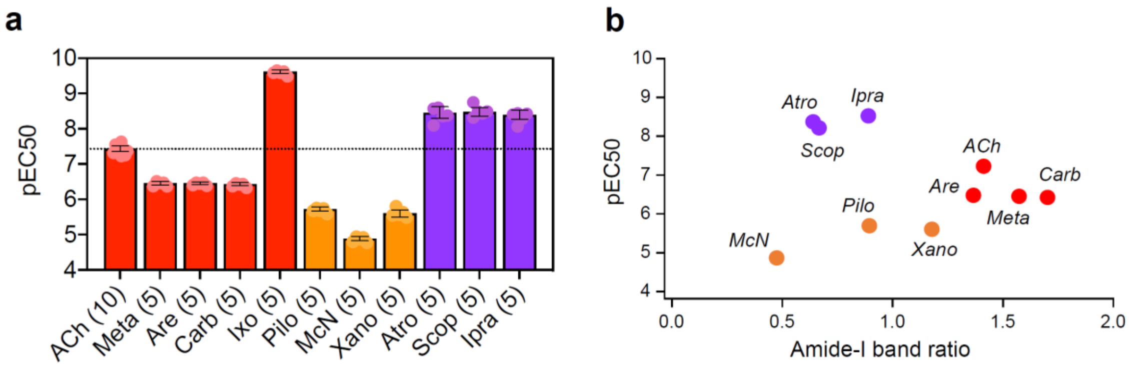

Supplementary Figure 7. (a) Potency of the different M_2R ligands toward G_i activation in the NanoBiT G-protein dissociation assay. pEC50 values were calculated from the concentration-response sigmoidal curves in Supplementary Figure 6. Bars and error bars represent mean and SEM, respectively, of 5 or 10 (shown in parenthesis) independent experiments with each performed in duplicate. (b) Plot of the amide-I percent population for agonists, partial agonists, and antagonists against their potency toward G_i activation in the NanoBiT G-protein dissociation assay. The amide-I band ratio values were calculated by the equation of efficacy rate, which was derived from Figure 2c, and pEC50 values were derived from (a). Agonists (ACh, Meta, Are, and Carb) were shown as red circles, partial agonists (Pilo, McN, and Xano) as orange circles, and antagonists (Atro, Scop, and Ipra) as purple circles.

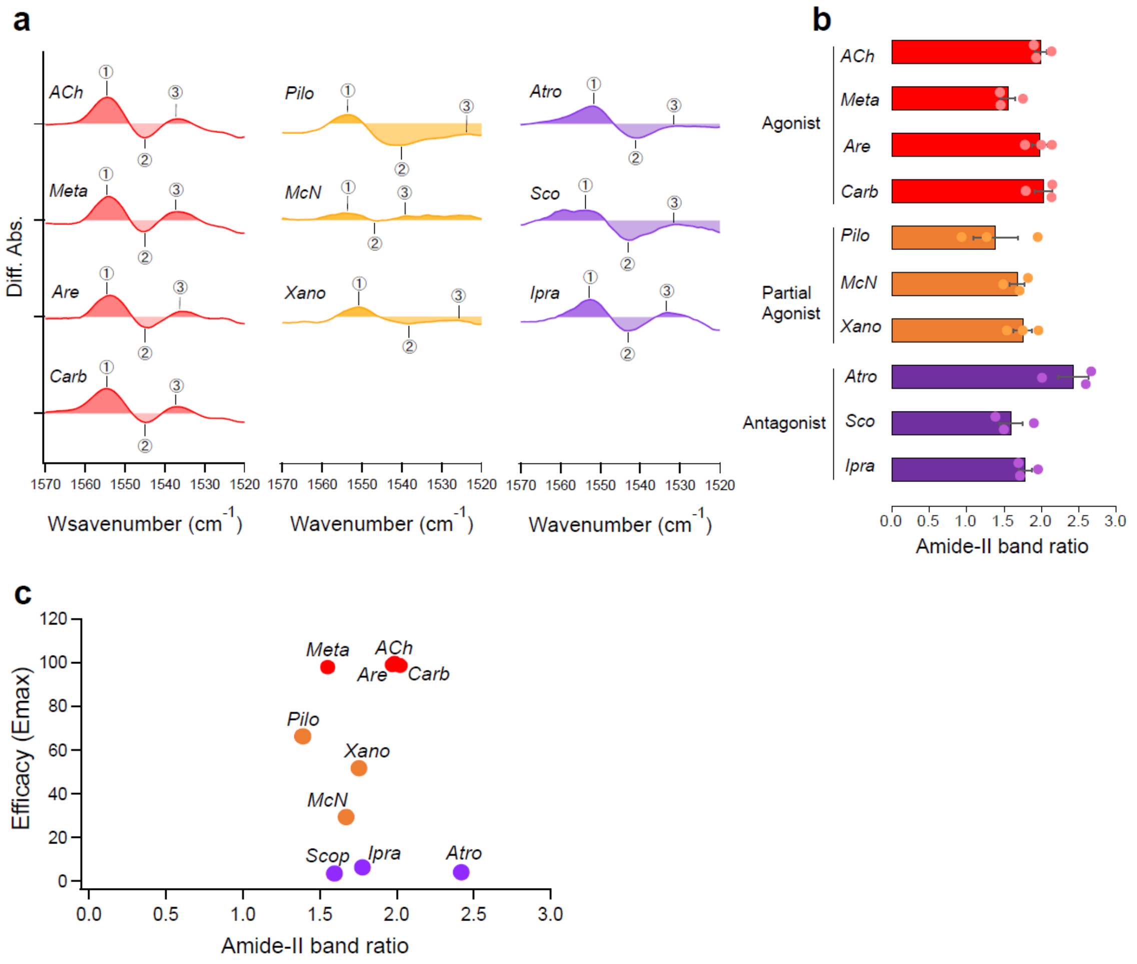

Supplementary Figure 8. (a) Ligand-dependent spectral changes of amide-II band originating from a coupled of the N-H in-plane bending and C-N stretching vibrations in 1570-1520 cm^{-1} region. Red, orange, and purple lines correspond to agonist-, partial agonist-, and antagonist-bound spectra, respectively. One division of the y-axis corresponds to 0.002 absorbance unit. The error values were calculated from three replicate experiments. (c) Correlation between ligand efficacy and the relative intensities of the amide-II bands. The relative intensities of the amide-II bands are calculated by the equation of efficacy rate, which is derived from (b). Ligand efficacy is determined by the NanoBiT G-protein dissociation assay from Fig. 3a. Agonists (ACh, Meta, Are, and Carb) are shown as red circles, partial agonists (Pilo, McN, and Xano) as orange circles, and antagonists (Atro, Scop, and Ipra) as purple circles.

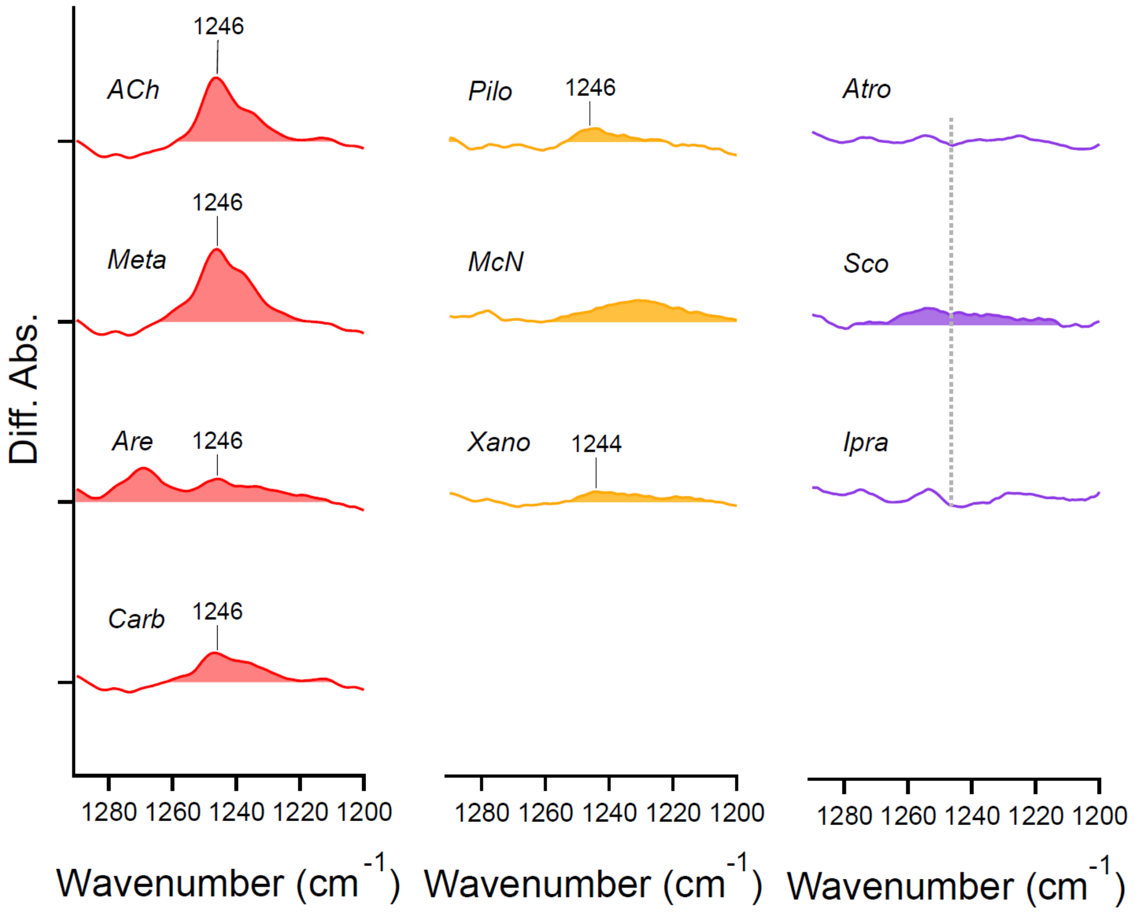

Supplementary Figure 9. Ligand-dependent spectral changes of the possible C-O stretch of tyrosine lid (Tyr104$^{3.33}$, Tyr403$^{6.51}$, and Tyr426$^{7.39}$) in 1290-1200 cm^{-1} region. Red, orange, and purple lines correspond to agonist-, partial agonist-, and antagonist-bound spectra, respectively. One division of the y-axis corresponds to 0.002 absorbance unit.

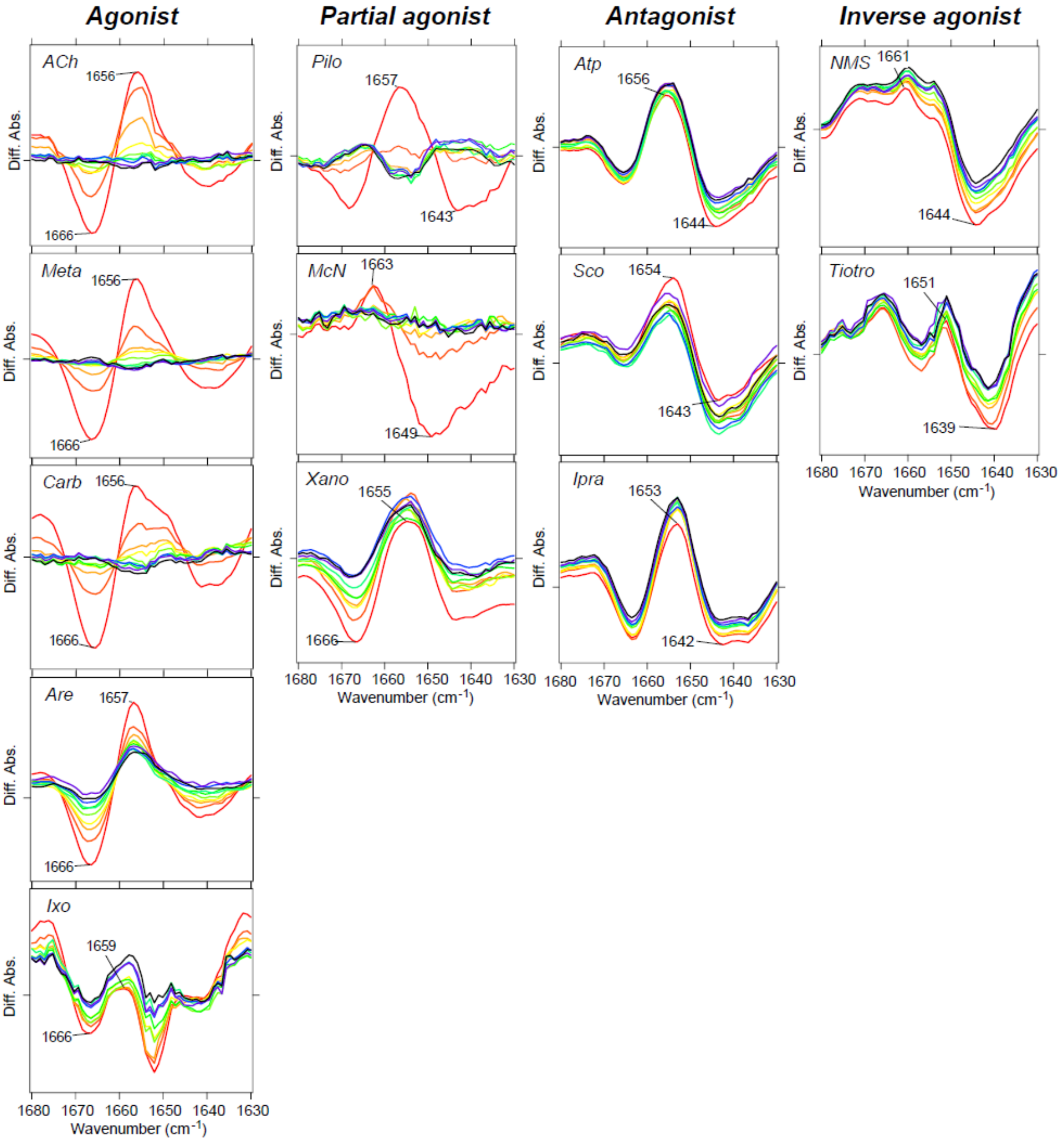

Supplementary Figure 10. Time-dependent difference ATR-FTIR spectra upon agonist (1st panel), partial agonist (2nd panel), antagonist (3rd panel) and inverse agonist (4th panel) dissociating in the amide-I (at 1680-1630 cm^{-1}) region.

ligand	k_{off} (min^{-1})
ACh	0.13 ± 0.02
MCh	0.18 ± 0.03
Carb	0.17 ± 0.02
Are	0.005 ± 0.001
Ixo	0.020 ± 0.001
Pilo	0.16 ± 0.05
McN	0.15 ± 0.02
Xano	0.010 ± 0.001
Atro	0.003 ± 0.000
Sco	0.002 ± 0.000
Ipra	N.D.*
NMS	0.010 ± 0.006
Tio	0.003 ± 0.000

*Indicates no dissociation from M_2R at least in the experimental conditions used in this study.

Supplementary Figure 11. Dissociation kinetics of each ligand from its receptors. Dissociation rate constants are expressed in per minute rate. Data are means ± S.E.M. from 3 independent experiments. *Indicates no dissociation from M_2R at least in the experimental conditions used in this study.

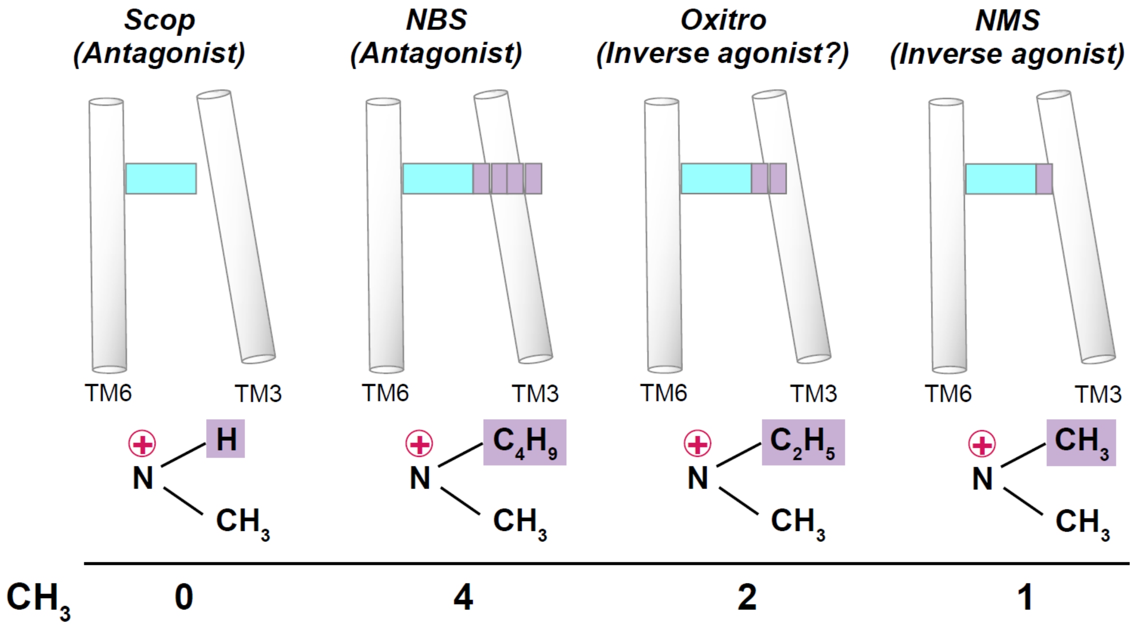

Supplementary Figure 12. Schematic drawing of binding fashion between antagonist and inverse agonist. Pale purple squares indicate the different moiety in tropane alkaloid among each ligand; Scopolamine (Scop), N-butylscopolamine (NBS), Oxitropium (Oxitro), and N-methylscopolamine (NMS). Pale cyan squares indicate the common chemical moiety among each ligand. Only NMS can fit the length between TM3 and TM6, which adopts an energetically favourable binding fashion to M_2R resulting in reducing the receptor activity at negative value.
