# Supplementary material for: Vibrational spectroscopy analysis of ligand efficacy in human M2 muscarinic acetylcholine receptor (M2R)
Source: Commun Biol. 2021 Nov 23;4:1321. doi: 10.1038/s42003-021-02836-1 (PMC8635417; doi:10.1038/s42003-021-02836-1)
Supplement: Supplementary file 3 — Reporting Summary [file 42003_2021_2836_MOESM3_ESM.pdf]

nature research

Corresponding author(s): Kota Katayama

Last updated by author(s): Sep 14, 2021

Reporting Summary

Nature Research wishes to improve the reproducibility of the work that we publish. This form provides structure for consistency and transparency in reporting. For further information on Nature Research policies, see our Editorial Policies and the Editorial Policy Checklist.

Statistics

For all statistical analyses, confirm that the following items are present in the figure legend, table legend, main text, or Methods section.

n/a	Confirmed	
☐	☒	The exact sample size (*n*) for each experimental group/condition, given as a discrete number and unit of measurement
☒	☐	A statement on whether measurements were taken from distinct samples or whether the same sample was measured repeatedly
☒	☐	The statistical test(s) used AND whether they are one- or two-sided<br>*Only common tests should be described solely by name; describe more complex techniques in the Methods section.*
☒	☐	A description of all covariates tested
☒	☐	A description of any assumptions or corrections, such as tests of normality and adjustment for multiple comparisons
☒	☐	A full description of the statistical parameters including central tendency (e.g. means) or other basic estimates (e.g. regression coefficient) AND variation (e.g. standard deviation) or associated estimates of uncertainty (e.g. confidence intervals)
☒	☐	For null hypothesis testing, the test statistic (e.g. *F*, *t*, *r*) with confidence intervals, effect sizes, degrees of freedom and *P* value noted<br>*Give P values as exact values whenever suitable.*
☒	☐	For Bayesian analysis, information on the choice of priors and Markov chain Monte Carlo settings
☒	☐	For hierarchical and complex designs, identification of the appropriate level for tests and full reporting of outcomes
☒	☐	Estimates of effect sizes (e.g. Cohen's *d*, Pearson's *r*), indicating how they were calculated

Our web collection on statistics for biologists contains articles on many of the points above.

Software and code

Policy information about availability of computer code

Data collection	Resolutions Pro Software for data acquisition of FTIR spectral collection.
Data analysis	Igor Pro8 for FTIR data. GraphPad Prism9 for G-protein activity assay data.

For manuscripts utilizing custom algorithms or software that are central to the research but not yet described in published literature, software must be made available to editors and reviewers. We strongly encourage code deposition in a community repository (e.g. GitHub). See the Nature Research guidelines for submitting code & software for further information.

Data

Policy information about availability of data

All manuscripts must include a data availability statement. This statement should provide the following information, where applicable:

- Accession codes, unique identifiers, or web links for publicly available datasets
- A list of figures that have associated raw data
- A description of any restrictions on data availability

Data supporting the findings of this manuscript have been deposited in Figshare.com and are available from the corresponding author upon reasonable request.

Field-specific reporting

Life sciences study design

All studies must disclose on these points even when the disclosure is negative.

Sample size	Sample sizes were determined based on prior literatures and best practices in the field; no statistical methods were used to predetermine sample size. The sample size for FTIR spectroscopy is 3-10 as indicated N number.
Data exclusions	No data were excluded.
Replication	All attempts at replication were successful.
Randomization	No randomization was applied.
Blinding	No binding was applied.

Reporting for specific materials, systems and methods

We require information from authors about some types of materials, experimental systems and methods used in many studies. Here, indicate whether each material, system or method listed is relevant to your study. If you are not sure if a list item applies to your research, read the appropriate section before selecting a response.

Materials & experimental systems

n/a	Involved in the study	
☐	☒	Antibodies
☐	☒	Eukaryotic cell lines
☒	☐	Palaeontology and archaeology
☒	☐	Animals and other organisms
☒	☐	Human research participants
☒	☐	Clinical data
☒	☐	Dual use research of concern

Methods

n/a	Involved in the study	
☒	☐	ChIP-seq
☒	☐	Flow cytometry
☒	☐	MRI-based neuroimaging

Antibodies

Antibodies used	mouse monoclonal antibody that specifically binds to the C-terminal epitope of rhodopsin (UBC Flintbox)
Validation	Validated by the manufacture. https://ubc.flintbox.com/technologies/0f1ef64b-fa5d-4a58-9003-3e01f6f672a6

Eukaryotic cell lines

Policy information about cell lines

Cell line source(s)	Sf9 cells were purchased from Thermo Fisher Scientific
Authentication	Cells have been authenticated by the vendors
Mycoplasma contamination	Cells were not tested for mycoplasma contamination.
Commonly misidentified lines (See ICLAC register)	Cells are not listed in the database.
